# The Personalization of Clopidogrel Antiplatelet Therapy: The Role of Integrative Pharmacogenetics and Pharmacometabolomics

**DOI:** 10.1155/2017/8062796

**Published:** 2017-03-21

**Authors:** Arwa M. Amin, Lim Sheau Chin, Dzul Azri Mohamed Noor, Muhamad Ali SK Abdul Kader, Yuen Kah Hay, Baharudin Ibrahim

**Affiliations:** ^1^School of Pharmaceutical Sciences, Universiti Sains Malaysia, Penang, Malaysia; ^2^Cardiology Department, Hospital Pulau Pinang, Penang, Malaysia

## Abstract

Dual antiplatelet therapy of aspirin and clopidogrel is pivotal for patients undergoing percutaneous coronary intervention. However, the variable platelets reactivity response to clopidogrel may lead to outcome failure and recurrence of cardiovascular events. Although many genetic and nongenetic factors are known, great portion of clopidogrel variable platelets reactivity remain unexplained which challenges the personalization of clopidogrel therapy. Current methods for clopidogrel personalization include* CYP2C19* genotyping, pharmacokinetics, and platelets function testing. However, these methods lack precise prediction of clopidogrel outcome, often leading to insufficient prediction. Pharmacometabolomics which is an approach to identify novel biomarkers of drug response or toxicity in biofluids has been investigated to predict drug response. The advantage of pharmacometabolomics is that it does not only predict the response but also provide extensive information on the metabolic pathways implicated with the response. Integrating pharmacogenetics with pharmacometabolomics can give insight on unknown genetic and nongenetic factors associated with the response. This review aimed to review the literature on factors associated with the variable platelets reactivity response to clopidogrel, as well as appraising current methods for the personalization of clopidogrel therapy. We also aimed to review the literature on using pharmacometabolomics approach to predict drug response, as well as discussing the plausibility of using it to predict clopidogrel outcome.

## 1. Introduction

Clopidogrel is a second-generation thienopyridine antiplatelet drug which exerts its effect by the inhibition of the platelet's purinergic receptor P2Y12 preventing adenosine diphosphate (ADP) from stimulating it. Clopidogrel is crucial drug for patients enduring high platelets reactivity such as coronary artery disease (CAD), acute coronary syndrome (ACS), and stroke. Some patients may require invasive therapy such as percutaneous coronary intervention (PCI) with stent placed in the occluded artery to ensure enough blood flow through it [1]. PCI patients have to take loading dose of clopidogrel prior to procedure followed by postprocedure dual antiplatelet therapy (DAPT) of low dose aspirin and clopidogrel for duration up to 12 months based on stent type and risk assessment [2]. This DAPT therapy is pivotal to prevent stent thrombosis (ST) and recurrence of ischemic events after PCI. However, some patients may suffer from attenuated platelets inhibition to clopidogrel or clopidogrel high on treatment platelets reactivity (HTPR) which hinders achieving the optimum outcome of DAPT. There are genetic and nongenetic factors contributing to clopidogrel HTPR, however, often challenging therapeutic outcome prediction [3]. Current methods of predicting clopidogrel response do not predict clopidogrel therapeutic outcome adequately. Therefore, investigating new approaches to assess clopidogrel response can help to achieve the desired outcome after PCI. In this review, we aimed to review the literature on clopidogrel variable platelets reactivity and appraise current methods to assess the clopidogrel therapeutic outcome. We also aimed to review the literature on new approaches such as pharmacometabolomics and integrative pharmacometabolomics-pharmacogenetics in assessing clopidogrel therapeutic outcome.

## 2. Clopidogrel Bioactivation and Clopidogrel HTPR

Clopidogrel is an oral drug which has oral bioavailability of 50% and the maximum peak concentration will be observed within 1 to 2 hours after the administration of the loading dose (600 mg) [4, 5]. The half-life of clopidogrel is from 7 to 8 hours [6]. Almost 50% of clopidogrel dose is excreted in the urine and 46% in the faeces [7]. Of the oral dose, approximately 85% is hydrolysed by esterases into inactive metabolite while the remaining 15% will be activated by the hepatic cytochrome P450 (CYP_450_) enzymes to the active metabolite through two steps of bioactivation [8]. The hepatic CYP_450_ enzymes which are involved in the bioactivation process of clopidogrel include the CYP1A2, CYP2B6, and CYP2C19 in the first step and the CYP2B6, CYP2C9, CYP3A4/5, and CYP2C19 in the second step [9–11]. The CYP2C19 enzyme plays vital role in the two bioactivation steps of clopidogrel by participating with 44.9% in the first step and 20.6% in the second step [9, 12]. The CYP3A4 has an essential role in the second step by participating with 39.8% [9]. Clopidogrel has minimum neutropenic side effect compared to ticlopidine (first-generation thienopyridine) [13]. The main side effects of clopidogrel are bleeding, gastrointestinal disorders, and rash, as well as other side effects such as hepatotoxicity and thrombotic thrombocytopenic purpura, albeit they are rare. Therefore, it is well tolerated by patients.

Patients variable platelets inhibition while on clopidogrel was first reported by Järemo et al. in 2002 [14]. In that study, five out of the eighteen PCI patients had weak platelets inhibition in response to clopidogrel loading dose of 300 mg. Since it was first reported, clopidogrel HTPR has been largely documented. It was found to be affecting 15–40% of the patients [4, 15, 16]. Clopidogrel HTPR is associated with poor outcome after PCI. Matetzky and colleagues indicated an association between clopidogrel HTPR and the risk of cardiac events' recurrence among 60 ACS patients undergoing PCI who had taken loading dose of 300 mg followed by daily dose of 75 mg for three months [17]. Substantiating these findings, Geisler et al. (2006) indicated that the primary end point of myocardial infarction, stroke, and death were significantly increased in clopidogrel HTPR patients who were followed up for three months after the PCI [18].

## 3. Genetic Factors Contributing to Clopidogrel HTPR

There are several identified genetic variabilities which contribute to clopidogrel HTPR. In fact, clopidogrel variable platelets reactivity is highly heritable [19]. As clopidogrel undergoes intestinal absorption, bioactivation by the hepatic CYP_450_ enzymes, and deactivation by esterases, this process might be affected by several genetic variabilities. Genetic variabilities which may interfere with clopidogrel variable platelets reactivity include the polymorphisms of the* CYP2C19* [19], the* CYP3A4/5* [20], the* CYP2C9* [21], the ATP-Binding Cassette Subfamily B Member 1* (ABCB1)* [22], the Paraoxonase-1* (PON1)*, the Carboxyl Esterase 1* (CES1*) [23], and the genetic polymorphism of the* P2Y12* receptors [24].

There are 57 active genes of the CYP_450_ enzymes and 58 pseudogenes in the human genome [25]. For most of the clinically relevant CYP_450_ enzymes, they are highly affected by genetic polymorphisms [26]. The CYP_450_ genetic polymorphisms could interfere with gene transcription, gene expression regulation, protein translation, and affinity to substrate [26, 27]. Among the* CYP*_450_ reported genetic polymorphisms, the* CYP2D6*,* CYP2C9,* and the* CYP2C19* were extensively studied and reported, respectively [28]. Of all studied* CYP*_450_ genetic polymorphisms, the* CYP2C19* polymorphisms were found to be highly associated with clopidogrel variable response, particularly that the enzyme is involved in the two bioactivation steps.

### 3.1. CYP2C19 Polymorphisms

The CYP2C19 enzyme plays substantial role in the metabolism and bioactivation of drugs such as proton pump inhibitors (PPIs) (omeprazole, lansoprazole, and pantoprazole), selective serotonin reuptake inhibitors (citalopram and sertraline), tricyclic antidepressant (imipramine and amitriptyline), phenytoin, and clopidogrel. The CYP2C19 enzyme is known by its classical reaction; the (S)-mephenytoin 4′-hydroxylation [29]. The gene of the* CYP2C19* is highly polymorphic with more than 25 alleles having variable enzymatic activity levels [30]. The star one allele (*∗*1) is the normal or “wild type,” a homozygous form of this allele's (*∗*1/*∗*1) phenotypes for the gained full function of the enzyme. Of all polymorphic alleles of the* CYP2C19*, two reduced functions alleles were identified as (*∗*2) and (*∗*3) alleles have been extensively studied. De Morais et al. (1994) had found that they cause poor metabolism of (S)-mephenytoin metabolism among Japanese [29]. Both the* CYP2C19∗2* and* CYP2C19∗3* alleles are single nucleotide polymorphism (SNP) of guanine (G) to adenine (A) present at nucleotide 681 in exon 5 and at nucleotide 636 in exon 4, respectively [29, 31]. The* CYP2C19∗2* causes aberrant splice site leading to the formation of stop codon which produces truncated, nonfunctional protein which is catalytically inactive [29]. The* CYP2C19∗3* causes premature termination codon which leads to the formation of truncated, nonfunctioning protein which is also catalytically inactive [31]. The* CYP2C19∗17* is another allele of the* CYP2C19* which is associated with increased gene transcription that leads to increased activity of the enzyme [32]. Clinically, individuals are classified into extensive metabolizers (EM), intermediate metabolizers (IM), poor metabolizers (PM), and ultrametabolizer (UM) of drugs based on their* CYP2C19* genotype [10].  Table 1 shows subjects'* CYP2C19* genotype and their CYP2C19 phenotype. The presence of at least one allele of the* CYP2C19∗2* is observed in 15–30%, 33–40%, and 40–50% of the Caucasians, African Americans, and Asians, respectively [33–35]. However, the* CYP2C19∗3* allele is present in less than 1% of both Caucasians and African Americans and 7% of Asians.

The importance of the* CYP2C19* loss of function (LoF) alleles,* CYP2C19∗2* and* CYP2C19∗3* effect on clopidogrel response, stems not only from their association with clopidogrel HTPR, but also from their association with poor therapeutic outcome [36]. Poor metabolizers (based on* CYP2C19* genotype) had lower plasma concentration of clopidogrel active metabolite [21]. Shuldiner and colleagues had found significant association between the* CYP2C19∗2* genotype and both the low platelets' inhibition to clopidogrel and the increase of cardiovascular events among PCI patients [19]. Mega and colleagues reported low level of the active metabolite of clopidogrel, reduced platelets inhibition to clopidogrel, and higher risk of ischemic events among patients who are carriers of the* CYP2C19* LoF alleles [34]. These results, as well as other findings, encouraged the United States (US) Food and Drug Administration (FDA) to issue labelled warning on clopidogrel box recommending the genotyping of* CYP2C19* in order to prevent recurrence of cardiovascular events [12].

### 3.2. Other Genetic Polymorphisms

Apart from the* CYP2C19* polymorphisms, the current literature is consistent about the limited effect of other* CYP* genetic polymorphisms on the exposure to clopidogrel active metabolite and platelets inhibition. Nevertheless, some controversy exists about some genetic variants. Brandt and colleagues investigated the effect of* CYP* polymorphisms on the exposure to clopidogrel active metabolite and platelets reactivity [21]. They indicated that the presence of the* CYP2C19* or the* CYP2C9* LoF variants was associated with reduced exposure to clopidogrel active metabolite and decreased platelets inhibition. However, the* CYP1A2*, the* CYP2B6*, the* CYP3A4,* and* CYP3A5* did not have any effect. Park and colleagues indicated similar findings; however, they did not investigate the effect of* CYP2C9* variants [37]. Although several studies showed that the* CYP2B6* polymorphism did not have an effect, a study indicated that carriers of the* CYP2B6* had lower exposure to clopidogrel active metabolite and lower platelets inhibition compared to noncarriers [34]. Although several studies suggested that the association between the* CYP3A4* and the* CYP3A5* polymorphisms and clopidogrel HTPR is not significant [34, 37–39], others indicated significant association between them [11, 20, 40]. This might be due to the major role of the CYP3A4 and CYP3A5 enzymes in the second step of clopidogrel bioactivation which may substantiate the effect of their genetic polymorphisms on clopidogrel exposure.

The* ABCB1* gene is the coding gene for the P-glycoprotein multidrug resistance-1 (MDR1) intestinal transporter, a modulator of clopidogrel absorption [41]. A genetic polymorphism of the* ABCB1* causes reduced absorption of clopidogrel [22]. Simon et al. (2009) found that ACS patients who were treated with clopidogrel and were carriers of the two variants alleles of the* ABCB1* had higher rate of cardiovascular events at one year of follow-up [35]. Similarly, Mega et al. (2010) indicated that the* ABCB1* polymorphism is significantly associated with the risk of deaths due to cardiovascular events or stroke among ACS patients treated with clopidogrel in the “Trial to Assess Improvement in the Therapeutic Outcomes by Optimizing Platelet Inhibition with Prasugrel-Thrombolysis in Myocardial Infarction” (TRITON-TIMI 38) trial [42]. However, other studies failed to indicate the same findings [19, 41].

As the majority of clopidogrel dose is being metabolized by the CES1 enzyme, a reduced function genetic polymorphism of this enzyme can lead to variable response to clopidogrel [23, 43]. Zhu et al. (2013) found that the inhibition of CES1 enzyme and the* CES1* reduced function genetic polymorphisms are associated with the reduced hydrolytic metabolism of clopidogrel and the increased concentrations of clopidogrel active metabolite in an in vitro model [43]. Lewis et al. (2013) studied the effect of the* CSE1* reduced function genetic variant on clopidogrel exposure and platelets inhibition among 566 healthy volunteers from the Amish “Pharmacogenomics of Anti-Platelet Intervention” (PAPI) study. The study revealed that the level of clopidogrel active metabolite is significantly higher among carriers of the reduced function allele [23]. In addition, the platelets inhibition measured by an ADP-stimulated platelet aggregation was higher in those who were carriers of the reduced function allele. Such findings suggest that the* CES1* reduced function genetic polymorphisms may play vital role in the variable platelets inhibitory effect of clopidogrel. Thus, it would be valuable for future investigations to test this role on patients' outcome.

The PON1 is an esterase enzyme which is synthesized in the liver [44]. The PON1, full enzymatic activity, is associated with atheroprotective effect which can be due to its role in enhancing an increased level of the high-density-lipoprotein (HDL) [45–47]. Therefore, patients who were carriers of the* PON1* lower activity genetic polymorphism were found to be having an increased oxidative stress and higher risk of cardiovascular events [45]. A presumed role of the PON1 enzyme in the metabolism of clopidogrel and its responsiveness stemmed controversy [46]. Bouman et al. (2011) used an in vitro metabolomics profiling which indicated that the PON1 is imperative enzyme in the hydrolytic cleavage of the intermediate metabolite of clopidogrel (2-oxo-clopidogrel) to the active metabolite of clopidogrel [44]. Accordingly, the* PON1* genetic variant of lower enzymatic activity causes lower level of clopidogrel active metabolite. Based on their findings, they conducted a case-cohort study on CAD patients undergoing PCI and a further prospective replication study on another independent sample of 1982 patients with ACS [44]. Both the case-cohort and the prospective replication studies concluded that there was an association between the genetic variant of* PON1* and the pharmacokinetics, the pharmacodynamics, and therapeutic outcome of clopidogrel [44]. These findings were challenged by other studies which indicated that this association is not significant [46, 47]. In fact, the effect of* PON1* genetic variant on the therapeutic outcome was attributed to the association between this variant and the risk of developing cardiac events [46, 47].

The data on the effect of the* P2Y12* genetic variants on clopidogrel response is dialectical. Some studies supported an effect of the* P2Y12* polymorphism on platelets activation and clopidogrel response [24, 48, 49], while others refuted this effect [50, 51] or indicated a synergistic effect of it, if coexisted with other genetic variabilities such as* CYP2C19* and* MDR1* [52, 53]. There are two haplotypes of the* P2Y12* gene (H1 and H2) where the H2 represents a gain-of-function haplotype of the receptor which might cause an increase in atherothrombosis and interfere with the response to clopidogrel [48]. The homozygous haplotype of H2 was found to be associated with clopidogrel HTPR [24].

Although the data are conflicting, it could be inferred from current literature that, except for the* CYP2C19*, most of the investigated genetic polymorphisms have weak or insignificant effect on the clopidogrel exposure and platelets inhibition. However, the coexistence of polymorphisms may have significant effect on platelets inhibition. In fact, the possession of the* CYP2C19* polymorphism and other polymorphisms can lead to synergistic effect. For instance, Mega and colleagues concluded that PCI patients who have both* ABCB1* and* CYP2C19* genetic variants may be vulnerable to the recurrence of cardiac events while they are on clopidogrel treatment [42]. Similarly, some studies indicated that coexistence of* CYP2C19* and* P2Y12* receptor genetic polymorphisms has higher effect on clopidogrel responsiveness and the clinical outcome than single polymorphism [52, 53]. Furthermore, the use of a drug with enzymatic inhibitory effect in patients who have polymorphism of reduced enzymatic activity for the same enzyme may lower platelets inhibition to clopidogrel. Park et al. (2013) indicated that patients on calcium channel blockers (CCBs) who were carriers of the* CYP3A4 (IVS 10 + 12) A *allele* (GA and AA)* had higher platelets reactivity while on clopidogrel compared to the noncarriers-wilt type* (GG)* [54].

## 4. Nongenetic Factors Contributing to Clopidogrel HTPR

Other multiple nongenetic factors interfere with clopidogrel response. Clinical factors such as concomitant diseases, drug-drug interaction, patient's compliance, obesity, and age are known to influence clopidogrel response. In addition, lifestyle factors such as smoking and diet can also affect the response.

The presence of comorbid condition such as type 2 diabetes mellitus (T2-DM) increases platelets reactivity in CAD patients which may cause poor therapeutic outcome [55]. In fact, T2-DM patients were found to endure clopidogrel HTPR while on DAPT [56]. However, the interindividual clopidogrel variable platelets reactivity among CAD patients with T2-DM might be due to the concomitance of genetic or nongenetic factors [55, 57]. It had been suggested that the administration of high maintenance dose of clopidogrel or more potent P2Y12 antagonists such as prasugrel can be used to overcome the clopidogrel HTPR in T2-DM [58]. Similar to T2-DM, patients suffering from chronic kidney disease (CKD) had reduced platelets inhibition to clopidogrel [59]. The decreased platelets inhibition in CKD patients was indicated for both clopidogrel and aspirin in patients on DAPT [60]. This reduced response is suggested to be due to the increased baseline platelets reactivity in CKD patients [60, 61]. The association between renal insufficiency and the risk of death and cardiac events after PCI is well established [62]. However, inconsistent data is available, in terms of the risk of bleeding, among PCI patients with underlying CKD [63, 64]. Noteworthily, the concomitance of comorbidities such as T2-DM with moderate to severe CKD was found to be associated with high platelets reactivity in CAD patients on DAPT which might lead to high frequency of poor outcome in this group of patients [65]. Recently, in Japanese population, it has been found that CKD is an independent predictor of PCI outcome, regardless of the* CYP2C19* genetic polymorphism status of the patient [66].

As clopidogrel is bioactivated by the CYP_450_ enzymes, it can be prone to drug-drug interactions, particularly drugs interfering with the CYP_450_ system. The PPIs are metabolized by the CYP_450_ enzymes; therefore, they might interact with clopidogrel [67]. However, the data on the extent of PPIs interact with clopidogrel remain controversial which suggests further investigation. In fact, the PPIs inhibit the CYP2C19 enzyme which might reduce the bioactivation of clopidogrel to its active metabolite [67, 68]. Accordingly, this could decrease the antiplatelet effect of clopidogrel which may increase cardiovascular events [68]. The PPIs may not interact to the same extent with clopidogrel [69]. In other words, some studies concluded that omeprazole was associated with the reduced antiplatelet effect of clopidogrel, while esomeprazole and pantoprazole were not [68, 69]. Similarly, lipophilic statins antihyperlipidemic drugs such as atorvastatin and simvastatin were reported to inhibit the antiplatelet effect of clopidogrel [70–72]. These statins interfere with the CYP3A4 enzyme [71]. Thus, it can mitigate the antiplatelet effect of clopidogrel. However, the preventive effect of statins by reducing lipid levels can halt cardiovascular events which might happen consequently to the reduced antiplatelet effect of clopidogrel [70]. Similar to the effect of statins albeit with some controversy in the literature, some drugs such as some CCBs, erythromycin, troleandomycin, and ketoconazole inhibit the CYP3A4 enzyme which will reduce the level of the clopidogrel active metabolite which can lead to reduced antiplatelet effect of clopidogrel [68, 73]. However, the data from some studies did not support this suggestion [74, 75]. For instance, the “Clopidogrel for the Reduction of Events During Observation” (CREDO) trial indicated that the concomitance use of the CCBs does not reduce the efficacy of clopidogrel and there was no evidence of interaction between them [75]. Anticoagulant drugs such as coumarin derivatives may also interfere with platelets inhibition by clopidogrel. Sibbing et al. (2010) indicated a significant reduction in the platelets' inhibitory effect of clopidogrel among patients concomitantly taking phenprocoumon when compared to those who are not taking phenprocoumon. This could be because of the interference with the capacities of the CYP3A4 and the CYP2C9 enzymes which are the main metabolizing enzymes of the phenprocoumon [76]. Sulfonylureas oral hypoglycaemic drugs may also reduce platelets inhibitory effect of clopidogrel [77]. This could be due to the interference with CYP2C9 enzyme, as well [77, 78]. On the other hand, there are also drugs which can increase or decrease the level of clopidogrel active metabolite by the inhibition or induction of the CES1 enzyme [43]. This might lead to clopidogrel variable platelets reactivity [79]. For instance, the phenobarbital, the dexamethasone, and the polycyclic aromatic hydrocarbons are drugs which can interfere with the activity of CES1 leading to variable levels of clopidogrel active metabolite [79].

Patient's compliance is crucial for achieving optimum therapeutic outcome. It has been found that noncompliance contributes significantly to clopidogrel HTPR [80]. Age, lipid levels, and body mass index (BMI) have been found to define nearly 22% of clopidogrel HTPR [19]. An age of more than 65 years old is associated with reduced platelets inhibitory effect of clopidogrel [81]. Furthermore, food, drinks, and smoking may interfere with clopidogrel response. For example, grapefruit can lower platelets inhibitory effect of clopidogrel which could be due to the interference with CYP_450_ isoforms [82]. In contrast, caffeinated drinks can increase platelets' inhibitory effect of clopidogrel [83]. Similarly, smoking was found to increase platelets' inhibitory effect of clopidogrel [84]. Smoking increases the bioactivation of clopidogrel to its active metabolite by the induction of the CYP1A2 and the CYP2B6 enzymes which are involved in the first step of the bioactivation of clopidogrel. This will increase platelets inhibition effect of clopidogrel.

## 5. Personalization of Clopidogrel Therapy

**Figure 1:**
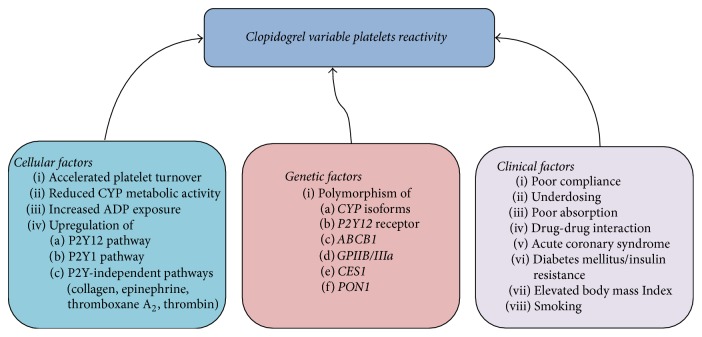
The genetic and nongenetic factors which may contribute to clopidogrel variable platelets reactivity. The figure presents the known genetic and nongenetic factors (cellular and clinical factors) which may contribute to clopidogrel variable platelets reactivity.* CYP*: cytochrome P450, ADP: adenosine diphosphate, GPIIB/IIIa: Glycoprotein IIB/IIIa, ABCB1: ATP-Binding Cassette Subfamily B Member 1. This figure is adapted and modified with permission from the publisher. Original source: Angiolillo and Ferreiro (2010) [85]. Copyright© (2010) Sociedad Española de Cardiología. Published by Elsevier España SL. All rights reserved.

It could be clearly understood that there are multifactorial genetic and nongenetic factors which interfere with clopidogrel variable platelets reactivity as depicted in Figure 1 [85]. However, these known factors failed to explain great portion of the variable platelets reactivity of clopidogrel [86]. For instance, Frelinger et al. (2013) assessed the pharmacokinetics and the pharmacodynamics of 75 mg daily dose of clopidogrel, administered by 160 healthy subjects for 9 days. The 160 subjects were free from nicotine for 6 weeks, prescription drugs for 4 weeks, over-the-counter drugs for 2 weeks, and caffeine and alcohol for 72 hours. All participants were homogenous* CYP2C19* EM genotype (*∗1/∗1)*. They were genotyped for the* ABCB1*,* PON1*, and* CYP3A5* polymorphisms. Of all participants, 45% had clopidogrel HTPR. Despite controlling the aforementioned factors, altogether factors such as age, weight, sex, platelet count, haematocrit, and genetic polymorphisms of* ABCB1*,* PON1*, and* CYP3A5* explained only 18% of the clopidogrel active metabolite peak plasma concentration (*C*_max_) and the area under the plasma concentration-time curve (AUC_*t*_) variabilities. When *C*_max_ and AUC_*t*_ were added to these factors, they explained 48% of pharmacodynamics variability. Noteworthily, the results showed that there was no significant association between the polymorphisms of* ABCB1*,* PON1*, and* CYP3A5* and the pharmacokinetics and pharmacodynamics of clopidogrel. These findings indicated the burden of the unexplained variability of clopidogrel response which consequently affects the therapeutic outcome of patients. This unsolved mystery can be due to unknown genetic polymorphisms or an interaction between several genetic polymorphisms and other nongenetic factors.

Personalized therapy is the use of person's genetics, proteomics, and environmental information to prevent, diagnose, and treat disease [87]. It aims to tailor patients therapy based on their genetic and nongenetic information rather than treating the whole population of a disease with the same treatment [88]. Since the discovery of clopidogrel variable platelets reactivity, it has been challenging to achieve optimum personalization of DAPT therapy. However, this optimum antiplatelet therapy could not be achieved without perfect prediction of the therapeutic outcome.

## 6. Adoption of Pharmacogenetics Biomarkers in Clinical Practice

The biomarker is a biological indicator of disease, physiological state, clinical status, response to drug therapy, or pathogenic process, which can be estimated and appraised for its indicative accuracy [89]. Accordingly, genetic variability which is associated with a biological status can be used as an indicative biomarker of that status. Pharmacogenetics biomarkers have been used to predict drug therapeutic outcome and avoid adverse drug reaction (ADR) prior to drug use. In 2008, the FDA issued table of valid pharmacogenetics biomarkers which contains list of drugs that had FDA label warning of pharmacogenetics testing prior to drug use and this list is frequently updated [90]. As there are several genetic factors which may interfere with clopidogrel variable platelets reactivity, genotyping of these polymorphisms was evaluated for clopidogrel outcome prediction [19, 34]. Apparently, the literature was consistent in this regard. The* CYP2C19* polymorphism predominates the effect of other genetic variants. Thus, the FDA considered the* CYP2C19* polymorphism valid pharmacogenetics biomarker of clopidogrel efficacy [90].

Although there are consistent literature asserting the association between clopidogrel HTPR and the* CYP2C19∗2* and *∗3* LoF alleles, in depth analysis of the data indicated that this association is strong in the PM who are carriers of the homozygous genotypes of the* CYP2C19 (∗2/∗2,∗3/∗3)* but not for the same extent with the IM who are carriers of the heterozygous genotypes (*∗1/∗2,∗1/∗3)* [4, 91–93]. Furthermore, patients who are EM but suffering from clopidogrel HTPR would be misclassified as responsive (having optimum clopidogrel platelets inhibition) based on their* CYP2C19* genotype [94]. Nasyuhana Sani and colleagues studied the effect of* CYP2C19* genotype on clopidogrel platelets reactivity among healthy volunteers from three East Asian ethnicities [92]. They had found that although carriers of one or two alleles of the* CYP2C19∗2* had significantly low platelets inhibition while on clopidogrel, low platelets inhibition was found in wild type homozygous carriers* CYP2C19 (∗1/∗1)* as well. Similarly, Mejin and colleagues had found that there was no significant association between clopidogrel HTPR and* CYP2C19* genotype among Malaysian CAD patients [93]. A systematic review and meta-analysis had concluded that, with the exception of stent thrombosis, the* CYP2C19* genotype is not significantly associated with cardiovascular events, despite its association with platelets aggregation [95]. Simply put, this can be merely justified by several facts. The* CYP2C19∗2* allele contributes to 12% of clopidogrel variable platelets reactivity which means great portion of this variability remains unpredicted by the genotype [19, 96]. The total activity of the CYP2C19 enzyme is affected not only by the genotype but also by the interacting inducer and inhibitor drugs [97]. In other words, some EM subjects may experience poor metabolizing activity of the CYP2C19 due to concomitant administration of inhibitor drugs and vice versa; the PM subjects taking concomitant inducer drugs may experience normal metabolizing activity of the CYP2C19. Besides, although it might be with considerably minimal effect, the synergetic effect of other genetic variabilities such as the* PON1*, the* CYP3A4/5*, the* ABCB1*, the* P2Y12* receptors, and the* CES1* cannot be neglected [20, 22–24, 40]. Indeed, it is expensive and less practical to genotype all the suspected clopidogrel related genetic variabilities. In addition, the limited role of genetic testing could be due to the fact that most of genetic mutations have inadequate prediction of the outcome [98]. As early mentioned, in the study of Frelinger et al. (2013), the known nongenetic and genetic factors explained 18% of the clopidogrel pharmacokinetics (PK) and 32%–65% of clopidogrel pharmacodynamics (PD). This means great portion of clopidogrel HTPR cannot be identified by* CYP2C19* genotyping. Therefore, the Clinical Pharmacogenetics Implementation Consortium Guidelines for* CYP2C19* genotype and Clopidogrel Therapy (CPIC), in its update (2013), did not recommend the* CYP2C19* guided therapy for all patients [10].

## 7. Pharmacokinetics Assessment of Clopidogrel Response

Several studies had evaluated different pharmacokinetics (PK) methods to determine the level of the parent compound clopidogrel and its metabolites as an indicator of clopidogrel platelets inhibition [5, 99, 100]. It was found that the level of clopidogrel active metabolite is correlated with clopidogrel platelets inhibition [86, 101], and the PK of clopidogrel could be predicted by the* CYP2C19* genotype [102]. However, applying PK to assess clopidogrel response is limited by several factors. The active thiol metabolite of clopidogrel is not stable which requires adding stabilizing agent within 30 seconds of blood sampling [101]. Some studies used indirect quantification of the active metabolite by quantifying the parent compound and the inactive metabolite [99, 103]. This means an accurate quantification of the active thiol metabolite in blood is difficult. Furthermore, the plasma level of the active metabolite of clopidogrel may not reflect the response in subjects affected by* P2Y12* receptor polymorphism, receptor density, fibrinogen, platelets concentration, and accelerated turnover of platelets variabilities [86, 104]. Therefore, the PK assessment can estimate the level of the parent compound and the active metabolite but it may not be optimum method to personalize clopidogrel antiplatelet therapy unless it is done together with other approaches such as pharmacogenetics or platelets function testing (PFT) to provide further information that can help to individualize the therapy.

## 8. Implementation of Platelets Function Testing (PFT) to Personalize Clopidogrel Therapy

Due to the limited prediction power of the pharmacogenetics biomarkers of clopidogrel platelets inhibition, the suggestion to personalize antiplatelet therapy based on platelets function testing (PFT) gained attraction. This had increased the demand to evaluate the use of PFT assays in the assessment of clopidogrel therapeutic outcome. PFT is one of the important methods to assess the effect of antiplatelet drugs and platelets defects. The PFT assessment of antiplatelet drugs relies on the measurement of platelets inhibition which happened due to the effect of the drug. There are different PFT instruments which are widely used to assess antiplatelet drugs such as the optical light transmission aggregometry (LTA) [105], the VerifyNow® system (VN) (Accumetrics Inc., San Diego, California) [106], the vasodilator stimulated phosphoprotein phosphorylation assay (VASP-P) [107, 108], the multiple electrode platelet aggregometry (MEA) [105], the platelets function analyser (PFA-100) [109–111], the Impact-R assay [110], and the platelet-works® (PW) [110]. The PFT instruments have different techniques, agonist reagents, and testing kits to test different classes of antiplatelet drugs [105, 109]. The PFT agonist reagent induces platelets activation in the sample through stimulation of the drug target. The aggregation of the platelets will be measured by the instrument and it indicates the status of platelets activity after the effect of the drug.  Table 2, compares some of the available PFT instruments. For each PFT instrument, there is a specified kit or agonist reagent to test clopidogrel response; it mainly contains ADP as stimulant of P2Y12 receptors. The LTA is the gold standard PFT assay [109]. However, the VASP-P assay is highly specific to P2Y12 receptor. Therefore, it is often considered the gold standard PFT assay for P2Y12 inhibitors. Several studies evaluated the ability of PFT assays to predict major cardiac events after PCI [112, 113]. However, the “Do Platelet Function Assays Predict Clinical Outcome in Clopidogrel-Pretreated Patients Undergoing Elective PCI” (Popular) study had compared the predictability of five different PFT assays [114]. The PFT assays were the LTA, VN, IMPACT-R, PW, and PFA-100. The study concluded that LTA, VN, and PW are the best PFT assessment predictors of atherothrombotic events including stent thrombosis, despite the limitation of low power in predicting bleeding risk. Generally, the use of PFT to tailor clopidogrel therapy has its limitations. The researchers from the “Double Randomization of a Monitoring Adjusted Antiplatelet Treatment versus a Common Antiplatelet Treatment for DES Implantation, and Interruption versus Continuation of Double Antiplatelet Therapy” (ARCTIC) clinical trial had concluded that tailoring antiplatelet therapy based on PFT did not improve outcomes [115]. This can be explained by several confounding factors. For many of PFT assays, the results are interfered by age, gender, ethnicities, diet, platelets count, and haematocrit which lowers their accuracy [109, 110, 116–118]. The results of PFT instruments which utilize platelets aggregation technique for measurement are highly affected by concomitant use of other antiplatelet drugs such as GPIIb-IIIa antagonists [119]. In fact, the simulation nature of PFT by using agonist in an in vitro setting to stimulate platelets activation does not perfectly resemble the physiological process of platelets activation which is affected by many internal pathways [108]. This may lead to PFT results that do not reflect the actual platelets function of the patients. Although the LTA and VASP-P assays are considered gold standard methods to measure platelets function, they are not advocated in clinical practice because they are time consuming and require lab facility and expert handling [120]. On the other hand, the VN-P2Y12 assay and other automated point of care (POC) PFT assays are fast and easy, but they are considerably expensive which limits the routine use of them. Platelets reactivity changes overtime in clopidogrel treated patients which necessitates frequent measurements for precise PFT based personalized therapy [121]. Moreover, there is inconsistency in the identification of clopidogrel responsiveness when the prespecified cut-off points of PFT assays were compared. In a study aimed to evaluate the agreement on clopidogrel HTPR identification between three PFT assays, the LTA, the VASP-P, and the VN-P2Y12, a reduced agreement was reported between the three assays. However, when the PFT results were tested for correlation as continuous variables, there was correlation between the results [122]. This implied that identifying clopidogrel HTPR based on one PFT assay is not enough and may lead to imperfect therapeutic decision. This might be one of the factors that hinders tailoring antiplatelet therapy based on PFT.

## 9. The Role of Systems Biology in Personalized Medicine

**Figure 2:**
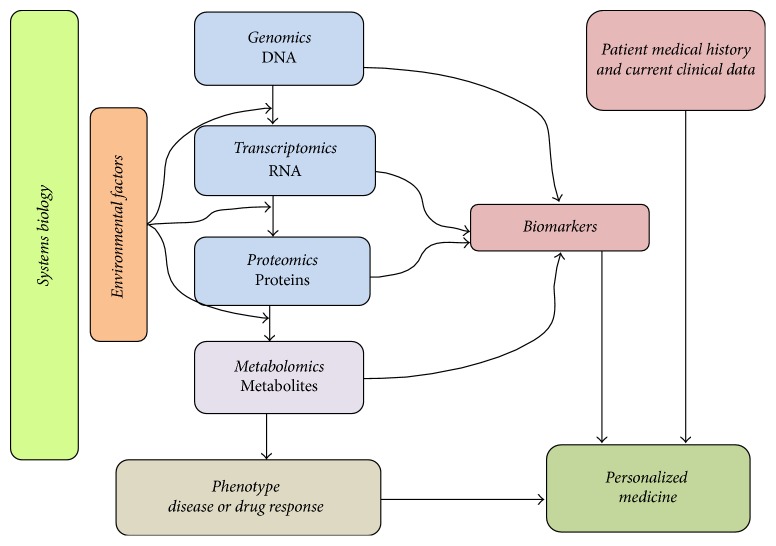
The role of Systems Biology in Personalized Medicine. The figure elucidates how all “Omics” disciplines can provide phenotype biomarkers (disease or drug response biomarkers). Together with patient medical history and current clinical data, these biomarkers can help to personalize patient's therapy. The metabolomics is the ultimate “Omics” that is close to the phenotype, as well as reflecting the perturbation in other “Omics.” This figure is adapted and modified with permission from the publisher. Original source: Louridas and Lourida (2012). Published by* Hippokratia*. All rights reserved [125].

Systems biology is the study of the biochemical, molecular, and supramolecular networks, along with their connections and interactions with the environmental factors in order to investigate an intricate biological perturbation of the living organism [123]. This term is an integrative approach which investigates not only the biological “Omics” such as genomics, transcriptomics, proteomics, and metabolomics, but also the environmental factors such as nutrition, gut microbiota, infections, other drugs, disease state, rest, and sleep which interfere with them. In fact, the conventional biology describes the biological processes in the living system without considering the connections and interactions between them and their environment; however, systems biology is comprehensive in terms of explaining the biological processes, their pathways networks, and other environmental factors which all together contribute to a phenotype such as disease or clinical trait [124, 125]. This will give an extensive understanding of that phenotype by using the multidisciplinary “Omics” data as biomarkers of the phenotype [125]. While the study of the genes and their association with the phenotype is the main interest of genomics, the study of the messenger ribonucleic acid (mRNA) and transcription process is the aim of transcriptomics [126]. The proteomics focuses on peptides and proteins expression [127]. Eventually, as all the substrates in the organism will be subjected to metabolism, the metabolomics focuses on the study of the metabolites [128]. The role of systems biology in the prediction of the phenotype and the personalization of therapy is shown in Figure 2.

Apart from pharmacogenomics biomarkers which had been extensively studied, there is scant data on the use of other systems biology approaches such as transcriptomics, proteomics, and metabolomics to find novel biomarkers of clopidogrel variable platelets reactivity and cardiovascular outcome. In a proteomics approach, Caruso et al., 2015, were the first to report an investigation of platelets proteomes after 24 hours of clopidogrel loading dose in 12 ACS patients (6 responders and 6 nonresponders). They indicated significant upregulation of the platelet adhesion molecule cluster differentiation-226 (CD226), downregulation of peroxiredoxin-4, and increased expression of transferrin in patients suffering from clopidogrel HTPR [129]. To the best of our literature review, there is no study which had investigated the use of metabolomics to assess clopidogrel response.

## 10. Disease and Drug Response Phenotyping Using Metabolomics

Metabolomics (metabonomics in some literature) is the study of the metabolites of the living organisms' metabolome [130]. The metabolites are the ultimate small molecules compounds which are outcomes of the metabolism of the systems biology components and the environmental factors interfering with it [131]. The metabolome of each living system varies with the variations in the systems biology components and the various environmental factors affecting it [130]. Accordingly, the metabolomes of subjects having particular phenotype (disease condition or drug response) cluster in discriminatory way from their controls upon analysing the metabolome of their biological samples [132]. This differentiation usually happens due to the presence of discriminating metabolites in the metabolome which is termed the metabolic fingerprint or metabotype [133]. The identified metabotype can be used as a biomarker of the phenotype. The metabolomics analysis involves analysing biological samples such as urine, serum, plasma, exhaled breath, sputum, cerebrospinal fluids (CSF), and cell culture using spectroscopic instruments such as nuclear magnetic resonance (NMR) or mass spectroscopy (MS) to identify and quantify the metabolites in the sample [134–136]. Metabolomics approach has been used in several studies to find novel biomarkers of diseases. It was used to identify new biomarkers of asthma, chronic obstructive pulmonary disease (COPD), cancer, and other diseases [135, 137, 138]. The use of metabolomics approach to explore and understand different cardiac conditions has been evolved. In an animal model of rats harbouring human renin and angiotensinogen genes, Mervaala and colleagues used metabolomics to test whether angiotensin II alters the metabolic profile of the heart [139]. They concluded that angiotensin II plays key role in the metabolic profile of the heart. Tenori and colleagues investigated the use of NMR profiling of serum and urine to find a metabolic finger print associated with heart failure (HF) and its relationship with New York Heart Association (NYHA) functional classes in humans [140]. The NMR analysis of serum and urine significantly discriminated HF patients from their healthy controls with high accuracy (greater than 86%). However, it was not able to discriminate patients among the NYHA classes of HF.

## 11. Pharmacometabolomics

Pharmacometabolomics or pharmacometabonomics in some literature is a metabolomics analysis which aims to discover novel biomarkers in the metabolome that predict drug response or toxicity [141, 142]. These novel biomarkers can be used as classifying tool to classify patients to responsive and nonresponsive to drug or may develop and may not develop drug toxicity [143]. Drug response metabotype not only predicts patient's response but also explains response related pathways and monitor patient's outcome during disease management which will improve the personalization of therapy [136, 143, 144]. The term was first proposed by Clayton et al., 2006. They analysed urine samples of rat pre-dose and postdose of 600 mg of paracetamol using proton nuclear magnetic resonance (^1^H-NMR) spectroscopy to find metabotype which is associated with paracetamol induced hepatotoxicity [145]. It was revealed that high level of predose taurine was associated with low liver damage assessed by mean histology score (MHS) [145]. However, predose low levels of trimethylamine-*N*-oxide (TMAO) and betaine were associated with increased paracetamol induced liver damage. A repeat of the study in healthy volunteers indicated that high level of predose urinary* p*-cresol sulfate was associated with low postdose urinary ratios of acetaminophen sulfate to acetaminophen glucoronate (S/G) which is due to the competence between acetaminophen and* p*-cresol on sulfotransferase enzyme [144]. Thus, the high level of endogenous* p*-cresol causes an increase in liver amenability for acetaminophen hepatotoxicity and its urine sulfate form can be used as predose predictive biomarker of it. The pharmacometabolomics studies have been evolved in using different biofluids samples to find metabotypes of drug efficacy and toxicity [136, 146–149]. In Table 3, the findings of some pharmacometabolomics studies in humans are summarized.

### 11.1. Pharmacometabolomics Spectroscopic Techniques

There are many spectroscopic methods commonly used in metabolomics/pharmacometabolomics studies such as nuclear magnetic resonance (NMR) or ^1^H-NMR, MS combined with gas or liquid chromatographic extraction (GC, LC) and Fourier transform infrared spectroscopy (FT-IR) [150–152]. Among them, ^1^H-NMR and MS are widely used [153]. Each of them has its advantages and limitations. The selection of the suitable spectroscopic method in a study depends on the features of each method and the compatibility of the method with the study's aim. The GC or LC combined MS method is more sensitive than ^1^H-NMR; thus it is highly recommended for metabolomics studies where particular group of compounds is being targeted such as lipid compounds [152, 154]. However, as it requires prior separation using LC or GC, it is time consuming and necessitates more lab work [145]. The ^1^H-NMR method is fast and does not require extensive sample processing [155]. Besides, prepared ^1^H-NMR samples can be run in large quantities with automated bulk run of samples [156, 157]. Samples prepared for ^1^H-NMR analysis can be stored and rerun for further analysis. Therefore, it is preferred choice for untargeted metabolomics analysis of large number of patients' samples.

### 11.2. Pharmacometabolomics Data Analysis

Pharmacometabolomics data analysis relies on prediction modelling using chemometrics analysis [158]. This includes multivariate analysis through the use of unsupervised and supervised machine learning methods [159]. The commonly used unsupervised method in metabolomics is the principal component analysis (PCA), while the supervised methods include methods such as partial least square (PLS), discriminant function analysis (DFA), and artificial neural networks (ANNs). The PCA provides an overview screening for the systematic variation of the data regardless of prior classification. Thus, it is exploratory analysis and usually followed by the supervised analysis where a prior input classification is introduced. The identification and pathway interpretation of the metabolites include combining knowledge in the pathophysiology of the phenotype with the analytical chemistry of the potential compound [160]. There are available databases which can be used to identify the putative biomarkers in metabolomics analysis such as Human Metabolome Database (HMDB), the Chenomx (Chenomx® Inc., Canada), the MetaboLights, the Birmingham Metabolite Library (BML-NMR), and the Biological Magnetic Resonance Data Bank (BMRB-metabolomics).

## 12. Pharmacometabolomics Informs Pharmacogenomics

The “pharmacometabolomics informs pharmacogenomics” approach implies that pharmacometabolomics can be used to identify genetic variation which is associated with the variation of drug response or toxicity [161]. Simply put, this concept is based on the fact that the variation in genes or genes' expression may lead to variations in proteins and eventually the metabolites levels which are associated with these pathways will change [162]. Accordingly, a metabotype which is associated with drug response may contain some metabolites associated with the variations in genes or genes expression which are implicated in the variable response. Ji and colleagues were the first to elucidate this concept on the variable response to the selective serotonin reuptake inhibitor (SSRI) escitalopram [163]. From major depressive disorder (MDD) patients on escitalopram whom were included in the Myo-PGRN SSRI study, 20 responders and 20 nonresponders were recruited for plasma pharmacometabolomics analysis. Baseline glycine levels were significantly different (*P* = 0.005) between the two groups and had negative association with the treatment outcome (remission) [163]. The researchers studied the glycine biosynthesis and metabolism to assign SNPs which are possibly related to these pathways. The selected SNPs were genotyped using the DNA of 512 patients from the same study (Myo-PGRN SSRI). Of the 135 assigned SNPs, the rs10975641 SNP from the glycine dehydrogenase gene was associated with response and remission. Similarly, using the same approach novel genetic variability associated with aspirin HTPR was discovered [164, 165]. Yerges-Armstrong et al. (2013) identified aspirin response metabotype in serum samples of healthy volunteers from the Heredity and Phenotype Intervention (HAPI) Heart study [164]. Aspirin HTPR group had significantly higher postaspirin levels of purine metabolites: inosine and adenosine. Association analysis of SNPs in the purine genes and aspirin HTPR led to the identification of the rs16931294 SNP in the adenosine kinase (ADK) gene region. Aspirin HTPR was significantly associated with the less common G allele rather than the more common A allele of the rs16931294 (*β* = 0.8, *P* = 0.00034). Furthermore, the G allele was significantly associated with preaspirin levels of adenosine monophosphate, hypoxanthine, and xanthine and postaspirin levels of inosine and guanosine. Therefore, pharmacometabolomics gained information may help to discover unknown genetic variations and response metabotypes can be used as an economical tool to predict both the response and the possible genetic cause of the response.

## 13. Conclusion

Clopidogrel had been well established as essential antiplatelet therapy paired with aspirin in DAPT therapy. Due to its high margin of safety, in terms of bleeding and side effects, and its price, clopidogrel remains the preferred P2Y12 antagonists in clinical practice. However, some patients fail to achieve therapeutic outcome while on clopidogrel. Finding precise method for predicting clopidogrel outcome is crucial to guide antiplatelet therapy. Current methods, the* CYP2C19* genotyping, PK, and PFT, have their drawbacks. Investigating other systems biology approaches such as proteomics and metabolomics may be the preeminence for clopidogrel personalization. Pharmacometabolomics approach has been used in assessing drug response, as well as identifying novel genetic variations associated with the response. As the perturbation in the metabolome is close to the phenotype, it can be highly reflective of drug's variable response. To date, pharmacometabolomics had never been evaluated in personalizing clopidogrel therapy. An integration of pharmacometabolomics and pharmacogenomics may not only tailor the therapy but also provide better understanding of the mystery behind clopidogrel HTPR.

## Figures and Tables

**Table 1:** Phenotype metabolising classes based on *CYP2C19* genotype^*∗*^.

Phenotype	Examples of the genotype	Enzyme activity
Ultrarapid metabolizer (UM)	*∗1/∗17*	Normal or increased enzyme activity
	*∗17/∗17*	

Extensive metabolizer (EM)	*∗1/∗1*	Normal enzyme activity

Intermediate metabolizer (IM)	*∗1/∗2*	Intermediate enzyme activity
	*∗1/∗3*	
	*∗2/∗17*	

Poor metabolizer (PM)	*∗2/∗2*	Low or absent enzyme activity
	*∗3/∗3*	
	*∗2/∗3*	

^*∗*^This table is adapted and modified with permission from the publisher. Original source: Scott et al. (2013) [10]. Copyright© (2013). Published by Wiley. All rights reserved.

**Table 2:** Platelets function testing devices.

PFT device	Specimen	PFT technique	Laboratory requirement/point of care (POC)	Agonist reagents/Cartridges
Light transmission aggregometry (LTA) [105]	Platelet rich plasma (PRP)	Optical light transmission aggregometry	Requiring trained lab staff and laboratory facility to do the test	ADP, arachidonic acid, epinephrine

Multiple electrode platelet aggregometry (MEA) [105]	Whole blood	Impedance aggregometry	Semiautomated	ADP, arachidonic acid, collagen

VerifyNow® system (VN) [106]	Whole blood	Optical light transmission aggregometry	Fully automated, POC	P2Y12 kit Aspirin kit GP IIb/IIIa kit

Platelets function analyser (PFA-100) [109–111]	Whole blood	High shear force dynamic flow system	Semiautomated, POC	Collagen with ADP Collagen with epinephrine

Impact-R [110]	Whole blood	Shear stress platelet adhesion	Requiring trained lab staff and laboratory facility to do the test. Extensive sample handling is needed	ADP

Vasodilator-stimulated phosphoprotein phosphorylation assay using flow cytometry (VASP-P) [107, 108]	Whole blood	Flow cytometric fluorescent quantification of intraplatelet VASP	Requiring trained lab staff, intensive lab work, sample preparation	VASP-P2Y12 assay

Platelet works (PW) [110]	Whole blood	Single platelet count	Semiautomated	ADP

POC: point of care, ADP: adenosine diphosphate, GP IIb/IIIa: glycoprotein IIb/IIIa, and VASP-P: vasodilator-stimulated phosphoprotein phosphorylation. P2Y12 kit: VerifyNow kit designed to test the P2Y12 receptor blockade to assess the response to P2Y12 inhibitors. Aspirin kit: VerifyNow kit designed to test the response to aspirin via arachidonic acid initiated reaction. GP IIb/IIIa kit: VerifyNow kit designed to test the response to GP IIb/IIIa inhibitors. VASP/P2Y12 assay: VASP phosphorylation correlates with the P2Y12 receptor inhibition.

**Table 3:** Examples of pharmacometabolomics studies in humans.

Study	Drug	Analytical method	Specimen	Main findings
Holmes et al., 2006 [136]	Antipsychotic drugs^*∗*^	^1^HNMR	CSF	A metabotype of schizophrenia which discriminated antipsychotic drug naive patients from healthy control. This metabotype was alleviated in half of the patients to normal after short term treatment with antipsychotic drugs.

Kim et al., 2010 [146]	Cyclosporine A (CsA) & Tacrolimus (TAC)	^1^HNMR	Serum	Time dependent metabolites changes in response to different treatment. The difference in the level of trimethylamine-*N*-oxide (TMAO) which is associated with graft dysfunction, between 2 groups of immunosuppressant drugs treatment; CsA & TAC were not significant. Lipid metabolites were higher in CsA group increasing the risk of cardiovascular diseases.

Wang et al., 2012 [147]	Methotrexate	^1^HNMR	Serum	Identification of 11 metabolites which can discriminate between methotrexate efficacy groups in patients with early rheumatoid arthritis.

Kaddurah-Daouk et al., 2013 [148]	Sertraline	GC-TOF-MS	Serum	Discriminating metabotypes of symptoms reduction between sertraline and placebo in MDD patients on one week and 4 weeks of treatment. Symptoms reduction after one week of sertraline treatment was associated with the reduction of 5-methoxytryptamine levels, while it was associated with lower levels of branched chain amino acids at four weeks of sertraline treatment.

Yerges-Armstrong et al., 2013 [164]	Aspirin	GC-MS	Serum	Identification of aspirin exposure metabotype of 18 metabolites in healthy volunteers who were on aspirin 81 mg for 14 days (HAPI study). Aspirin exposure metabotype was significantly associated with purine metabolic pathway. Inosine and adenosine were significantly higher after aspirin in aspirin HTPR group.

Villaseñor et al., 2014 [149]	Ketamine	LC-QTOF-MS	Plasma	Identification of discriminating metabolites between responders and nonresponders of ketamine among bipolar depression patients. The discriminating metabolites were related to mitochondrial *β*-oxidation of fatty acid.

Ellero-Simatos et al., 2014 [166]	Aspirin	LC-MS	Serum	Elevated level of baseline serum serotonin is associated with aspirin HTPR based on collagen induced PFT assessment of healthy subjects who were on aspirin 81 mg for 14 days (HAPI study).

^1^HNMR: proton nuclear magnetic resonance, GC: gas chromatography, TOF: time of flight, MS: mass spectroscopy, QTOF: quadrupole time of flight, LC: liquid chromatography, CSF: cerebrospinal fluid, CVDs: cardiovascular diseases, CsA: Cyclosporine A, TAC: Tacrolimus, TMAO: trimethylamine-*N*-oxide, MDD: major depressive disorder, HAPI: Heredity and Phenotype Intervention, and HTPR: high on treatment platelets reactivity.

^*∗*^ *Antipsychotic drugs: atypical antipsychotic drug such as amisulpride, clozapine, olanzapine, risperidone, quetiapine, and ziprasidone.*
